# Bilingualism, Autism, and Mental Health: Implications for Social Work

**DOI:** 10.7759/cureus.103129

**Published:** 2026-02-06

**Authors:** Hana Abbasian

**Affiliations:** 1 Center for Bioethics, Harvard Medical School, Boston, USA

**Keywords:** autism spectrum, bilingualism, cultural diveristy, mental health services, neurodiversity, social work practice

## Abstract

Bilingual children with autism grow up in families navigating complex social, cultural, and structural contexts. In addition to challenges common to many families, they may experience distinct stressors related to migration, minoritized language status, and navigating differences between home cultural norms and those of the dominant society. Language guidance in autism care refers to clinical and professional recommendations about family language use, including whether and how multiple languages are supported in home, educational, and therapeutic settings. Because language practices structure daily interaction, caregiving routines, and children’s access to relationships and community, such guidance can meaningfully influence family routines, emotional connection, and access to culturally grounded support networks that are important for mental health and well-being. This conceptual editorial, based on the existing interdisciplinary literature, examines bilingualism through a social work perspective, discussing how practitioners can support families in ways that respect both neurodiversity and cultural identity. Culturally responsive and intersectional approaches enable social workers to consider overlapping social identities, structural factors, and community networks that shape mental health outcomes. By facilitating informed family decision-making and integrating language guidance with broader community support, social workers can help families maintain connection, resilience, and agency. Considering bilingualism as a meaningful aspect of development allows social workers to support family well-being, promote equity, and provide community-based support in autism care.

## Editorial

Autism Spectrum Disorder (ASD) is a neurodevelopmental condition characterized by persistent difficulties in social communication and social interaction, and by repetitive patterns of behavior, interests, or activities [1]. The term "spectrum" reflects the wide variation in the type, severity, and manifestation of symptoms among individuals with ASD [1,2]. Language guidance in autism care refers to structured clinical recommendations and support provided to families to promote the children’s communication development and functional language use in everyday contexts [1,2]. Because early language development is closely linked to later social, cognitive, and educational outcomes, language guidance is widely recognized as one of the earliest and most important intervention targets in autism care [1,2]. Bilingualism and multilingualism refer to the regular use of two or more languages within the home or community environments in which a child is raised [1,2]. In many clinical contexts, bilingual families of children with autism may be advised to limit language exposure to a single dominant language to reduce perceived cognitive or communicative burden on the child [1,2]. Therefore, language guidance becomes one of the earliest and most important interventions that children with autism and their families receive [1]. Language guidance in autism is typically provided by multidisciplinary specialists, such as speech-language pathologists, social workers, psychologists, and early education professionals through direct therapy sessions, caregiver coaching, home visits, and structured parent training programs [1,2].

For bilingual families in marginalized communities, this guidance may arrive as a suggestion to simplify, streamline, or narrow linguistic exposure [2]. These recommendations are frequently framed as pragmatic or developmentally protective [2,3]. Within social work practice, language guidance carries ethical, relational, and structural implications that extend far beyond speech outcomes [3].

In bilingual and multilingual families, language operates as cultural infrastructure, meaning the everyday system through which values, caregiving practices, discipline, faith, and belonging are transmitted [2]. For children with autism, access to this infrastructure shapes social development in ways that clinical metrics alone may not capture [1]. Social workers should be aware of these dynamics in home visits, intake interviews, school meetings, and family counseling sessions.

Despite growing evidence showing comparable developmental outcomes among bilingual and monolingual children with autism, bilingualism may be framed through a deficit lens [2,4]. Through this lens, bilingualism becomes something to manage, reduce, or delay. These messages persist even as research demonstrates stable intact cognitive flexibility and social benefits associated with bilingual exposure [2,4].

For marginalized families, these recommendations land within an uneven power landscape [5]. Power asymmetry describes the imbalance between professionals who control access to diagnoses, services, and accommodations, and families who depend on these systems for support [5]. When language guidance is delivered from a position of authority, families may feel pressure to comply even when recommendations conflict with cultural values or caregiving realities. Families from immigrant and refugee backgrounds often rely on shared language as a stabilizing force that supports emotional regulation, caregiving continuity, and connection during periods of transition [1,2]. Within these contexts, language holds practical and relational value that extends into daily coping and family resilience. When guidance discourages home language use, families may experience added strain in already complex environments [2]. Social workers who attend to these broader conditions can situate language decisions within the realities of stress, adjustment, and mental health, supporting families in ways that reflect their full social context [2,3]. 

An intersectional perspective further informs social work engagement with bilingual children with autism and their families. Intersectionality describes how social identities such as disability, language, culture, and socioeconomic position coexist and shape lived experience [5]. For many families, these identities are navigated simultaneously within healthcare, education, and social service systems [1,3]. For example, a bilingual autistic child from a low income immigrant family may face multi-layered barriers related to disability, language access, cultural expectations around communication, and limited availability of linguistically appropriate services, which together shape how care recommendations are experienced and implemented.

Social workers are often trained to recognize how mental health experiences emerge through these overlapping contexts [3]. Applying an intersectional approach supports assessments and interventions that reduce oversimplified narratives and strengthen advocacy for supports that reflect the realities of diverse family lives.

Social work ethics emphasize understanding individuals within their social, cultural, and relational contexts [3]. Language practices form a central part of this environment [1]. When bilingualism is discouraged, families may experience cultural dissonance, the stress that arises when institutional expectations clash with lived identity [1,2]. This dissonance can reduce trust in services, increase disengagement, and create feelings of surveillance within healthcare and educational systems [3,5].

In the context of autism and bilingualism, culturally responsive social work includes asking how language functions in daily routines, discipline, storytelling, and expressions of care [2,3]. It includes sharing evidence about bilingual development in accessible terms and supporting families in making decisions that reflect both developmental goals and cultural continuity.

In school settings, social workers frequently participate in individualized education planning. Misconceptions about bilingualism may influence placement decisions, service eligibility, or expectations of progress [2,4]. Social workers can challenge assumptions that multilingual environments complicate learning. They can collaborate with educators to design supports that respect linguistic identity while addressing individual learning needs. Social workers can also collaborate with community leaders to disseminate accurate information about bilingualism and autism, countering myths through trusted channels. This approach improves collective knowledge and reduces isolation among families navigating similar decisions.

Respectful communication remains essential. Families may receive conflicting messages across clinical, educational, and social service settings. Social workers can serve as translators of systems, meaning they help families interpret recommendations, weigh evidence, and articulate their values [3]. This role supports informed consent, defined as making decisions with a clear understanding of options, risks, and benefits.

As autism care increasingly emphasizes equity and inclusion, bilingualism in marginalized communities deserves sustained attention. Social work practice grounded in cultural humility, evidence awareness, and community partnership can transform language guidance from a source of tension into an opportunity for empowerment. By affirming bilingualism as a legitimate and meaningful dimension of social development, social workers contribute to care models that honor both neurodiversity and cultural diversity. In moments of guidance, advocacy, and accompaniment, social work shapes how families experience belonging, dignity, and agency within systems that deeply affect their lives.

At the policy level, clinical guidelines and early intervention frameworks should move beyond monolingual defaults by explicitly supporting bilingual and multilingual development in autism care, alongside investments in linguistically accessible services and culturally responsive provider training. Future research should prioritize longitudinal and community-engaged studies that compare different models of language guidance and examine their developmental, relational and psychosocial effects on autistic children and their families. Such work is particularly needed in immigrant and marginalized communities, where language practices intersect with structural inequities in access to care.
